# Differences in Hypothalamic Lipid Profiles of Young and Aged Male Rats With Impaired and Unimpaired Spatial Cognitive Abilities and Memory

**DOI:** 10.3389/fnagi.2020.00204

**Published:** 2020-07-03

**Authors:** Judith Wackerlig, Harald C. Köfeler, Volker Korz, Ahmed M. Hussein, Daniel D. Feyissa, Harald Höger, Ernst Urban, Thierry Langer, Gert Lubec, Jana Lubec

**Affiliations:** ^1^Department of Pharmaceutical Chemistry, Faculty of Life Sciences, University of Vienna, Vienna, Austria; ^2^Center for Medical Research, Medical University of Graz, Graz, Austria; ^3^Department of Neuroproteomics, Paracelsus Private Medical University, Salzburg, Austria; ^4^Core Unit of Biomedical Research, Division of Laboratory Animal Science and Genetics, Medical University of Vienna, Vienna, Austria; ^5^Neuroscience Laboratory, Paracelsus Medical University, Salzburg, Austria

**Keywords:** learning, memory, lipids, hypothalamus, hippocampus

## Abstract

Lipids play a major role for several brain functions, including cognition and memory. There is a series of work on individual lipids showing involvement in memory mechanisms, a concise lipidome was not reported so far. Moreover, there is no evidence for age-related memory decline and there is only work on brain of young vs. aging animals. Aging animals, however, are not a homogeneous group with respect to memory impairments, thus animals with impaired and unimpaired memory can be discriminated. Following recent studies of hippocampal lipid profiles and hypothalamus controlled hormone profiles, the aim of this study was to compare hypothalamic, lipidomic changes in male Sprague-Dawley rats between young (YM), old impaired (OMI) and old unimpaired (OMU) males. Grouping criterions for aged rats were evaluated by testing them in a spatial memory task, the hole-board. YMs were also tested. Subsequently brains were removed, dissected and hypothalami were kept at −80°C until sample preparation and analysis on liquid chromatography / mass spectrometry (LC-MS). Significant differences in the amounts of a series of lipids from several classes could be detected between young and aged and between OMI and OMU. A large number of lipids were increased in OMI and a smaller number in OMU as compared to young rats. Differences of lipid ratios (log2 of ratio) between OMI and OMU consisted of glycerophosphocholines (aPC 36:2 and 36:3; PC 34:0, 36:1, 36:3 and 40:2); Glycerophosphoethanolamines (aPE 34:2, 38:5 and 40:5; LPE 18:1, 20:1, 20:4, 22:4 and 22:6; PE36:1 and 38:4); glycerophosphoserines (PS 36:1, 40:4, and 40:6); triacylglycerol TG 52:4; ceramide Cer 17:2 and sphingomyelin SM 20:0. Thus, hypothalamic lipid profiles across different lipid classes discriminate aged male animals into OMU and OMI. The underlying mechanisms may be related to different functional networks of lipids in memory mechanisms and differences in metabolic processes. The study underlines the importance of lipidomics in the pathophysiology of age-related cognitive decline. The necessity of evaluating the cognitive status of aged subjects by behavioral tests results in more specific detection of critical lipids in memory decline, on which now can be focused in subsequent memory studies in animals and humans.

## Introduction

Lipids play a pivotal role in the regulation of brain functions (Piomelli et al., 2007; Chan et al., 2012). A most useful review article (Morley and Banks, 2010) is reporting the role of lipids in cognition, stating that cholesterol, omega-3-fatty acids and triglycerides have been postulated to modulate cognition in humans, both, in health and disease. In this critical review it is also made clear that results from experimental treatments with lipids are not unequivocal, however, it is concluded that obesity as well as lipid loads by nutrition are linked to cognitive decline. Obesity and metabolic dysfunction related to age may also cause cognitive dysfunction *via* endothelial damage and the related dysregulation of cerebral blood flow, insufficiency of the latter has been widely accepted to cause cognitive impairment (Tucsek et al., 2014). Lipid consumption modulate blood pressure and cardiovascular – as well as cerebrovascular – function thereby contributing to cognitive performance (De Wilde et al., 2003).

So far, the lipid neurochemical basis has not been fully provided in studies related to cognition and memory. Mostly, the work is focusing on individual lipids although the introduction and development of mass spectrometric techniques with the aim to generate brain lipidomes has allowed deeper insight into lipid composition and mechanisms in several species and a series of brain subregions and subareas (Köfeler et al., 2010; Oliveira et al., 2016; Smidak et al., 2017b; Lerner et al., 2019; Maliković et al., 2019b).

Some reports have been addressing the effect of aging and cognitive decline on the brain lipidome (Hopiavuori et al., 2017; Šmidák et al., 2017a). Very few have considered the fact that there are aging rodents with preserved memory or with memory decline (Yetimler et al., 2012). The fact that the factor age does not simply represent a homogenous group has been already addressed in previous publications clearly indicating differences of neuronal functions and gene expression in aged subjects (Brouillette and Quirion, 2008; Ménard and Quirion, 2012).

In previous studies we focused on differences in hippocampal lipid profiles in good and bad learners (Maliković et al., 2019a) as well as steroid and thyroid hormone levels between young male rats, comparable in terms of spatial memory performance to OMU and OMI male rats selected from a large cohort by a spatial memory paradigm, the hole-board (Maliković et al., 2019b). These hormones are generally related to spatial learning and memory by modulating hippocampal plasticity (McGaugh et al., 1975; Bohus and de Kloet, 1981; Joëls and de Kloet, 1991; Joëls, 2018) and the release and production are widely regulated by the hypothalamus. We found the Thyroid-stimulating hormone (TSH) to be the only one to discriminate between OMU and OMI. The hypothalamic region is involved in memory processes (Chamorro-López et al., 2015; Albert-Gascó et al., 2017). Lee et al. (2012) found decreased levels of nuclear but not cytosolic levels of the glucocorticoid receptor (GR) in the hippocampus of aged cognitively impaired rats which was related to dysfunctions of the Hypothalamus-pituitary-adrenal axis (HPA) (Lee et al., 2012). Nuclear steroid receptors act as transcription factors and are involved in slow long-term memory related processes (McGaugh et al., 1975; Bohus and de Kloet, 1981; Joëls and de Kloet, 1991; Joëls, 2018). Through the medial septum/diagonal band there is also a direct connection between the hypothalamus and the hippocampus that mediate spatial learning (Albert-Gascó et al., 2017). Differences in hypothalamic lipids could be related to aging and memory (Yetimler et al., 2012; Das, 2018). Thus, differences in the generation of lipidomes in this brain region is potentially important for linking lipid levels or patterns to age-related cognitive decline. However, so far little information on a comprehensive lipid composition of this area has been shown.

Therefore, the present study aimed to determine differences in the hypothalamic lipidome between YM, OMI and OMU rats. Because the hormone study has been done by using the hole-board we chose this spatial leaning paradigm also for the present study.

## Materials and Methods

### Animals

#### Keeping

Animals were bred and maintained in the Core Unit of Biomedical Research, Division of Laboratory Animal Science and Genetics, Medical University of Vienna. The animals lived in a separate experimental room 1 week before and throughout the experiment. Rats were housed individually in standard Makrolon cages filled with autoclaved woodchips at a temperature of 22 ± 2°C with a humidity of 55 ± 5% and 12 h artificial light/12 h dark cycle (light on at 7:00 am). The study was carried out according to the guidelines of the Ethics Committee, Medical University of Vienna, and was approved by the Federal Ministry of Education, Science and Culture, Austria.

### Behavioral Testing

Three groups of male Sprague–Dawley rats (10 animals per group) were tested for their lipid profile in the hypothalamus, namely YM, OMI and OMU rats. YM rats were 3 months old with an average weight of 440 g, old male rats were 20 months old with an average weight of 650 g. In order to distinct between old male rats with impaired and unimpaired memory, a hole-board memory test was performed according to a previously described protocol (Smidak et al., 2017b). The spatial memory performance was evaluated based on the reference memory index (RMI) calculated as following: (first + revisits of baited holes)/total visits of all holes. The individuals were considered as good performers when RMI > (the mean RMI value + standard deviation), and as bad performers when RMI < (the mean RMI value – standard deviation), with the mean value obtained from all animals included in the analysis over the evaluated training/testing period. For the rats considered in this study the mean RMI during trial 10 was OMU: 0.97 ± 0.09; OMI: 0.24 ± 0.29. Body weights were not different between OMU (504 g ± 71.5 g) and OMI (542 g ± 44.5 g) with *t* = -1.44 and *p* = 0.17. To determine statistical significance of the differences over the entire training between all groups of rats, the data were analyzed by SPSS Version 22 software (IBM^®^ SPSS^®^ Statistics) using general linear model – repeated measures ANOVA. In order to detect significant differences in single trials we performed two-group comparisons between all groups by using the *t*-test. The significance was set at *p* ≤ 0.05. The animals were decapitated, the brains were rapidly removed and dissected on a Para Cooler (RWW Medizintechnik, Hallerndorf, Germany) at 4°C to obtain the hypothalamus. The tissue was immediately stored at −80°C until lipidomic analysis.

### Analysis of Lipids

Brain lipids were extracted by a methyl-tert-butyl ether (MTBE) protocol as previously reported (Fauland et al., 2011) with 2.5 nmol PC 12:0/12:0 (Avanti Polar Lipids, Alabaster, AL, United States) added to each sample before extraction for monitoring the extraction efficiency. Briefly, dissected hypothalamus samples were put in 4 mL of methanol:MTBE (1:1.67 v/v) and homogenized on ice for 30 s by an Ultra-Turrax homogenizer. After adding 2.5 mL MTBE and 1.25 mL MS-grade water (for phase separation), the upper organic phase was taken off and the lower aqueous phase was re-extracted with 2 mL MTBE. The combined organic phases were dried and re-suspended in 1,000 μL methanol:chloroform (1:1 v/v). For analysis an aliquot of the former solution was again dried and re-suspended in 100 μL isopropanol:chloroform:methanol (90:5:5 v/v/v). Data acquisition was performed on an LTQ Orbitrap Velos Pro instrument (Thermo Scientific) coupled to a Dionex Ultimate 3000 UHPLC (Thermo Scientific) following previously published protocols (Fauland et al., 2011; Triebl et al., 2017). Briefly, chromatographic separation was performed on a Waters (Waters, Milford, MA, United States) BEH C8 column (100 mm × 1 mm, 1.7 μm), at 50°C with a flow rate of 150 μL/min. The mobile phase A was composed of deionized water containing 10 mM ammonium formate and 0.1 vol% of formic acid. The mobile phase B was a mixture of acetonitrile/isopropanol 5:2 (v/v) with 10 mM ammonium formate and 0.1 vol% of formic acid as well. Gradient elution started at 50% mobile phase B, rising to 100% B over 40 min with a washing phase at 100% B for 10 min and a final re-equilibration of the column with 50% B for 8 min before the next injection. Injection volume was 2 μL and samples were kept at 8°C in the autosampler until measurements. The mass spectrometer was operated in Data Dependent Acquisition mode using an HESI II ion source. Every sample was measured both in positive and negative polarity. Ion source parameters for positive polarity were: Source Voltage: 4.5 kV; Source Temperature: 275°C; Sheath Gas: 25 arbitrary units; Aux Gas: 9 arbitrary units; Sweep Gas: 0 arbitrary units; Capillary Temperature: 300°C. Ion source parameters for negative ion mode were: Source Voltage: −3.8 kV; Source Temperature: 325°C; Sheath Gas: 30 arbitrary units; Aux Gas: 10 arbitrary units; Sweep Gas: 0 arbitrary units; Capillary Temperature: 300°C. The automatic gain control target value was set to 106 ions to enter the mass analyzer, with a maximum ion accumulation time of 500 ms. Full scan profile spectra were recorded from m/z 400 ± 1,350 for positive and negative ion mode and were acquired in the Orbitrap mass analyzer at a resolution setting of 100,000 at m/z 400. For MS/MS experiments, the ten most abundant ions of the full scan spectrum were sequentially fragmented in the ion trap using helium as collision gas (CID, Normalized Collision Energy: 50; Isolation Width: 1.5; Activation Q: 0.2; Activation Time: 10) and centroided product spectra at normal scan rate (33 kDa/s) were collected. The exclusion time was set to 10 s.

### Data Analysis

Differential lipidomic analysis of YM, OMU and OMI rats were performed by SIEVE^TM^ software (version 2.1, Thermo Scientific) for peak detection and alignment into features. Subsequently, manual feature identification was performed. A twofold difference between groups was chosen as cut-off criterion, in order to reduce the high number of lipids to the most meaningful for statistical analysis. The lipids that reach this criterion were different between individual groups. Thus two group comparisons were conducted with the *t*-test with adjusted p-values for multiple comparisons. Lipids that reach the criterion in all three groups were tested by univariate ANOVA and (if significant) by subsequent post-hoc tests (Tukey-HSD) for between group comparisons. All tests were two-tailed. For evaluation of statistically significant data a *p*-value of *p* < 0.05 was chosen. Lipid species were annotated according to the Lipid MAPS shorthand nomenclature (Fahy et al., 2011; Liebisch et al., 2013) and are denotated as AA X:Y whereas AA represents the abbreviation of the lipid molecule in general and X is the total number of carbon atoms and Y the total number of double bounds in their acyl side chains. For each test group ten individuals were consulted. Level changes are obtained by the average ratio of the peak areas of lipid species in the three groups of YM/OMI, YM/OMU and OMU/OMI. All data is presented as base 2 logarithm of the respective ratio in order to facilitate interpretation of the level changes in lipid species. The respective standard error of the mean (SEM) was calculated for each group for all determinants.

## Results

### Behavior

**FIGURE 1:**
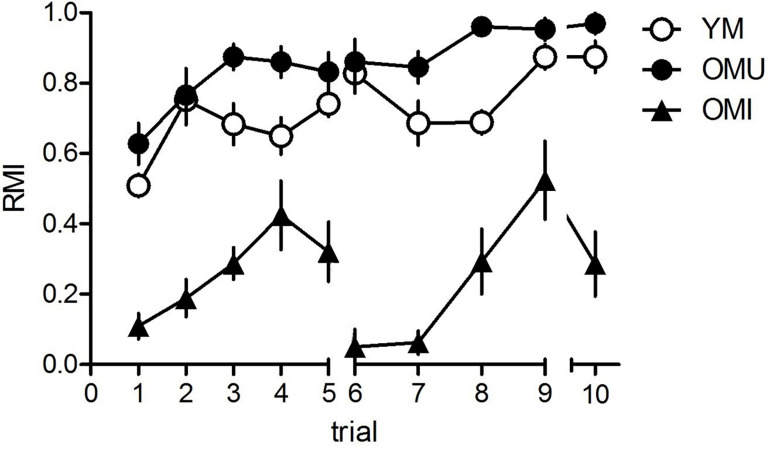
Behavioral results for the hole-board test. Young males (YM) performed better than old impaired males (OMI) showing higher reference memory indices (RMI) but not compared to old unimpaired males (OMI) whereas between YM and OMU there was no difference (for statistics see the section “Results”). Given are the mean values and standard errors of the mean, *n* = 10 for each group). Statistics: 10 animals per group; data analyzed by SPSS Version 22 software (IBM^®^ SPSS^®^ Statistics) using general linear model – repeated measures ANOVA; the significance cut-off was set at *p* < 0.05. Spatial memory performance was evaluated based on reference memory index (RMI) calculated as following: (first + revisits of baited holes)/total visits of all holes; good performers if RMI > (the mean RMI value + standard deviation); bad performers when RMI < (the mean RMI value – standard deviation); the mean value obtained from all animals included in the analysis over the evaluated training/testing period. The values are expressed as mean ± SEM.

The results of the hole-board tests are given in Figure 1. We found a significant trial effect (*F*_9,243_ = 9.11, *p* < 0.001), a significant trial × group interaction (*F*_18,243_ = 9.11, *p* = 0.001) and a significant effect between groups (*F*_2,27_ = 181.14, *p* < 0.001). There was no significant effect in single trials between YM and OMU except of trial 8 with significant higher RMI in OMU as compared to YM (*t* = 3.24, *p* < 0.05), whereas in all trials except trial 4 we found significant differences between YM and OMI (trial 1: *t* = 4.76, trial 2: *t* = 6.71, trial 3: *t* = 4.72, trial 5: *t* = 5.02, trial 6: *t* = 9.28, trial 7: *t* = 7.44, trial 8: *t* = 4.71, trial 9: *t* = 4.18, trial 10: *t* = 7.02; *p* < 0.05 each) with higher RMI in the first. Between OMU and OMI we found significant effects in all trials with higher RMI in OMU (trial 1: *t* = 7.51, trial 2: *t* = 6.23, trial 3: *t* = 10.02, trial 4: *t* = 4.09, trial 5: *t* = 5.07, trial 6: *t* = 9.94, trial 7: *t* = 13.85, trial 8: *t* = 6.93, trial 9: *t* = 3.71, trial 10: *t* = 7.08; *p* < 0.05 each).

Thus, OMU performed widely similar as YM, whereas OMU performed widely poorer than the both other groups.

### Lipids

In this study a high throughput non-targeted lipidomic analysis was performed in order to identify significant level changes in lipid species in the hypothalamus depending on aging and impaired memory of male rats. The dissected hypothalami of YM, OMU and OMI rats (10 individuals each) were analyzed by LC-MS with ESI-MS both in positive and negative mode. Lipid species were identified applying an internal lipid database covering more than 20,000 molecular lipid species from 58 individual lipid (sub)classes by their accurate (+/-5 ppm) precursor mass.

In total 120 lipid species were detected in the hypothalamus of rats during this study. However, 59 lipid species were found to be statistically significant (*p*-value <0.05) up-or downregulated for at least two times in at least one of the three groups (YM/OMI, YM/OMU and OMU/OMI). All lipid species with significant level changes in at least one of the three groups were detected in positive mode. Generally, the most significant and consistent level changes were observed in the group of YM/OMI (53 out of 59 lipid species). All identified lipid molecules with significant level changes are listed in Table 1 with their respective *p*-value and the respective log2 value of the ratio of the areas from YM, OMU and OMI rats forming the three groups. Lipid species are denoted as AA X:Y on the abbreviation of lipid molecule AA, the total number of carbon atoms (X) and the total number of double bonds (Y) in their acyl side chains. Seven lipids reached the cut-off criterion in all three groups and were tested by ANOVA. LPE 18:1: *F*_2,27_ = 29.46; LPE 20:4: *F*_2,27_ = 29.86; LPE 22:4: *F*_2,27_ = 32.71; LPE 22:6: *F*_2,27_ = 29.14; aPC 36:2: *F*_2,27_ = 14.91; PS 40:4: *F*_2,27_ = 15.06; PS 40:6: *F*_2,27_ = 23.55, *p* < 0.001, each. Post hoc tests revealed higher contents of YM as compared to both aged groups (*p* < 0.001, each) and higher levels in OMU as compared to OMI (*p* = 0.010) for LPE 18:1, LPE 20:4 (*p* < 0.001, each and *p* = 0.009, LPE 22:4 (*p* = 0.001, each, OMI vs. OMU *p* = 0.010), LPE 22:6 (YM vs. OMI *p* < 0.001, YM vs. OMU *p* = 0.001, OMI vs. OMU *p* = 0.008) and PS 40:6 (YM vs. OMI *p* < 0.001, YM vs. OMU *p* = 0.005, OMI vs. OMU *p* = 0.005). For PS 40:4 and aPC 36:2 higher contents in YM as compared to aged (YM vs. OMI *p* < 0.001, each; YM vs. OMU *p* = 0.007 and *p* = 0.005, respectively) could be detected. However, no differences in the levels of these lipids could be determined between the aged groups (*p* = 0.117 and *p* = 0.144, respectively).

**TABLE 1:** The summary of identified lipid species with statistically significant (adjusted *p*-values <0.05) level changes in the hypothalamus of the three groups of young male rats compared to old male rats with impaired and unimpaired memory.

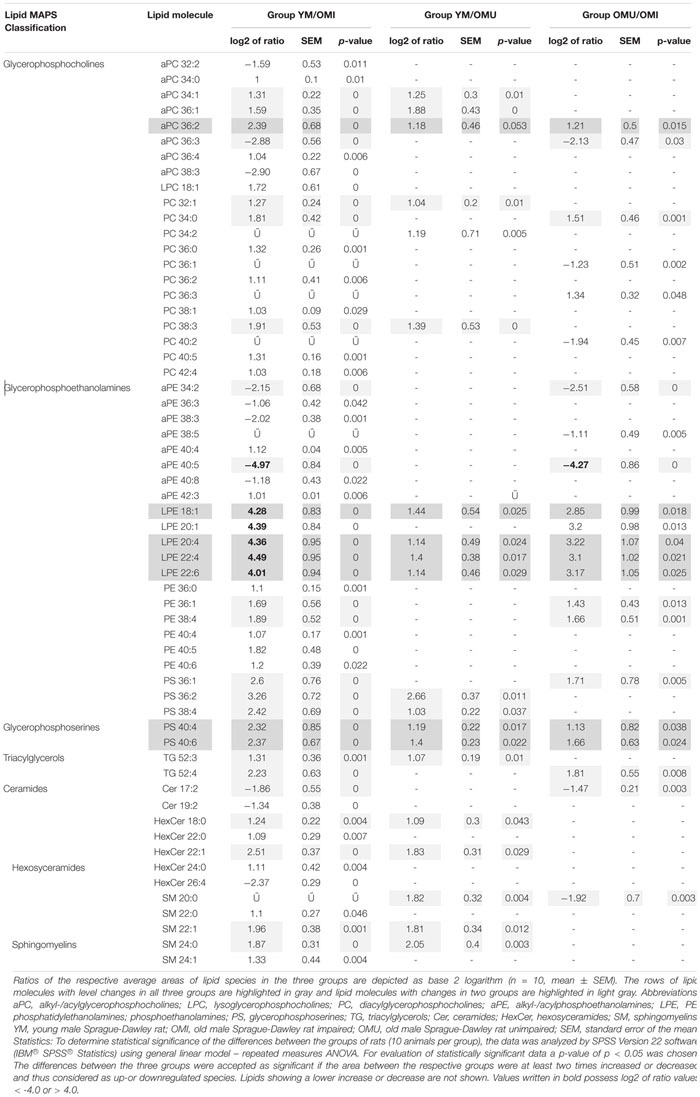

#### Glycerophospolipids

**FIGURE 2:**
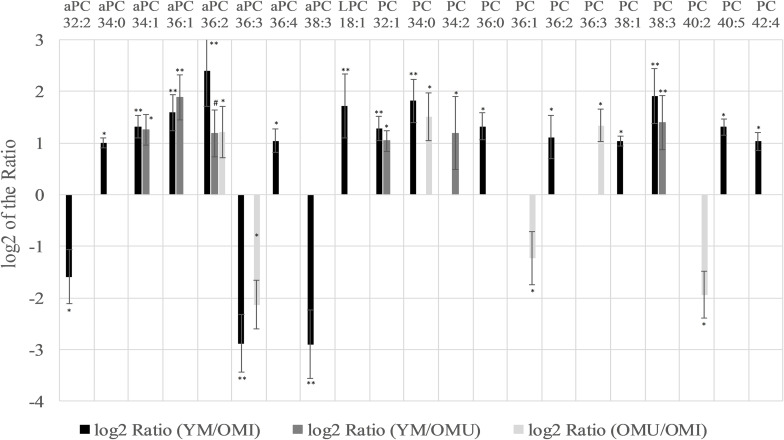
Significant level changes in the glycerophospholipid main class of glycerophosphocholines (aPC, LPC, and PC) in the hypothalamus with either up- or downregulated levels concerning aging and impaired memory. Abbreviations: aPC, alkyl-/acylglycerophosphocholines; LPC, lysoglycerophosphocholines; PC, diacylglycerophosphocholines; YM, young male Sprague-Dawley rat; OMI, old male Sprague-Dawley rat impaired; OMU, old male Sprague-Dawley rat unimpaired. Statistics: Differential lipidomic analysis of YM, OMU, and OMI rats were performed by SIEVE^TM^ software (version 2.1, Thermo Scientific) for peak detection and alignment into features followed by manual feature identification. For evaluation of statistically significant data a *p*-value of *p* < 0.05 was chosen. Values are marked as following: *p* < 0.05 (^∗^), *p* < 0.001 (^∗∗^) and in one case *p*-value >0.05 – even not significantly different, there was a trend (#). The differences between the three groups were accepted as significant if the area between the respective groups were at least two times increased or decreased, all other lipids are not further investigated. All data is presented as base 2 logarithm of the respective ratio. The respective standard error of the mean (SEM) was calculated for each group for all determinants. The lipid aPC 36:2 reaches the criterion in all three groups and was thus further tested by univariate ANOVA (*F*_2,27_ = 14.91) and by subsequent post-hoc tests (Tukey-HSD) for between group comparisons. All tests were two-tailed. For evaluation of statistically significant data a *p*-value of *p* < 0.05 was chosen. Post hoc tests revealed higher contents of YM as compared to both aged groups (*p* < 0.001., each). A higher content in YM as compared to aged male rats (YM vs. OMI *p* < 0.001, YM vs. OMU *p* = 0.005) could be detected. However, no differences in the levels of this lipid could be determined between the aged groups (*p* = 0.144).

Glyceriphospholipid (GP) changes were detected in the class of glycerophosphocholines with in total twenty-one out of forty-one detected lipid species showing statistically significant level changes (Table 1 and Figure 2). Five out of them are downregulated, all other glycerophosphocholines are upregulated in association with aging and impaired memory. In more detail, twelve out of twenty-eight diacylglycerophosphocholines (PC), eight out of eleven alkyl-/acylglycerophosphocholines (aPC) and one out of two lysoglycerophosphocholines (LPC) were determined as significantly changed in level. Generally, PCs were significantly increased in association with aging. However, PC 36:1 and 40:2 were significantly downregulated in OMU as compared to OMI. The level changes associated with impaired memory of aPCs were either up- or downregulated but did not show any correlation depending on chain length and saturation. aPC 34:1, 36:1 and 36:2 can also be associated with aging showing increased levels in YM compared to OMU. The level of one lyso species of PCs (LPC 18:1) was increased in YM compared to OMI.

**FIGURE 3:**
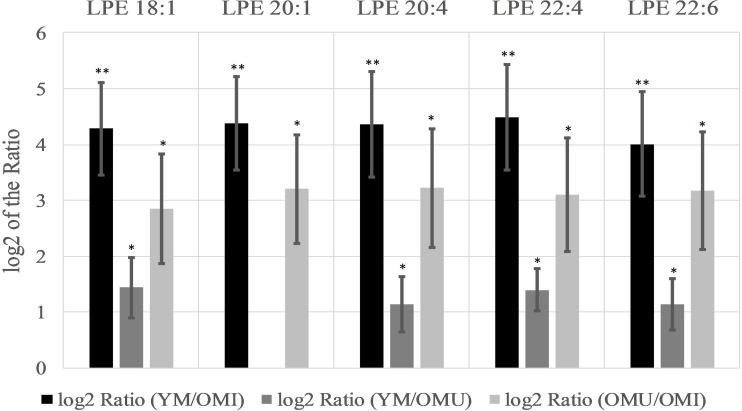
Significant level changes of LPE lipid species in the hypothalamus all showing increased log2 values concerning aging and impaired memory (*n* = 10, mean ± SEM). Abbreviations: LPE, phosphatidylethanolamines;YM, young male Sprague-Dawley rat; OMI, old male Sprague-Dawley rat impaired; OMU, old male Sprague-Dawley rat unimpaired;aPC, alkyl-/acylglycerophosphocholines; LPC, lysoglycerophosphocholines; PC, diacylglycerophosphocholines; YM, young male Sprague-Dawley rat; OMI, old male Sprague-Dawley rat impaired; OMU, old male Sprague-Dawley rat unimpaired. Statistics: Differential lipidomic analysis of YM, OMU and OMI rats were performed by SIEVE^TM^ software (version 2.1, Thermo Scientific) for peak detection and alignment into features followed by manual feature identification. For evaluation of statistically significant data a *p*-value of *p* < 0.05 was chosen. Values are marked as following: *p* < 0.05 (^∗^) and *p* < 0.001 (^∗∗^). The differences between the three groups were accepted as significant if the area between the respective groups were at least two times increased or decreased, all other lipids are not further investigated. All data is presented as base 2 logarithm of the respective ratio. The respective standard error of the mean (SEM) was calculated for each group for all determinants. Four out of five LPE lipids reached the criterion in all three groups and were thus further tested by univariate ANOVA (LPE 18:1: *F*_2,27_ = 29.46; LPE 20:4: *F*_2,27_ = 29.86; LPE 22:4: *F*_2,27_ = 32.71; LPE 22:6: *F*_2,27_ = 29.14) and by subsequent post-hoc tests (Tukey-HSD) for between group comparisons. All tests were two-tailed. For evaluation of statistically significant data a *p*-value of *p* < 0.05 was chosen. *Post hoc* tests revealed higher contents of YM as compared to both aged groups (*p* < 0.001, each) and higher levels in OMU as compared to OMI (*p* = 0.010) for LPE 18:1, LPE 20:4 (*p* < 0.001, each and *p* = 0.009, LPE 22:4 (*p* = 0.001, each, OMI vs. OMU *p* = 0.010) and LPE 22:6 (YM vs. OMI *p* < 0.001, YM vs. OMU *p* = 0.001, OMI vs. OMU *p* = 0.008).

Specifically, five lysoglycerophosphoethanolamines (LPE) showed the most significant changes of all analyzed lipids being upregulated with a log2 ratio in the YM group compared to OM between 4.0 and 4.5 (compare Figure 3). The log2 ratio between YM and OMU also showed upregulated values for four of the LPEs in YM compared to OM which were found to be between 1.1 and 1.4 in the mean and for the log2 ratio of OMU and OMI between 2.9 and 3.2. These findings indicate that the LPEs may act as markers for aging and impaired memory as well. In detail, three LPEs, namely LPE 18:1, 20:4 and 22:6, are upregulated in all three groups with the slightest increase in OMI and therefore may act as the most important biomarkers for memory on the one hand and age-related memory decline on the other hand. LPE 20:1 is not significantly changed between YM and OMU but may be considered as marker for impaired memory as it was significantly upregulated in YM and OMU as compared to OMI (see Table 1). LPE 22:4 seems to act as a marker for aging in general as there is no significant difference between OMU and OMI but both groups showed significantly decreased levels as compared to YM.

**FIGURE 4:**
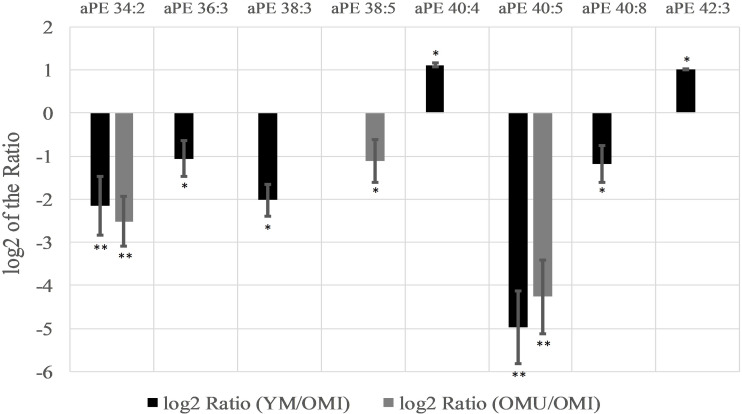
Significant level changes of aPE lipid species in the hypothalamus with either up- or downregulated levels in the groups YM/OMI and OMU/OMI. Abbreviations: aPE, alkyl-/acylphosphoethanolamines; YM, young male Sprague-Dawley rat; OMI, old male Sprague-Dawley rat impaired; OMU, old male Sprague-Dawley rat unimpaired;aPC, alkyl-/acylglycerophosphocholines; LPC, lysoglycerophosphocholines; PC, diacylglycerophosphocholines; YM, young male Sprague-Dawley rat; OMI, old male Sprague-Dawley rat impaired; OMU, old male Sprague-Dawley rat unimpaired. Statistics: Differential lipidomic analysis of YM, OMU and OMI rats were performed by SIEVE^TM^ software (version 2.1, Thermo Scientific) for peak detection and alignment into features followed by manual feature identification. For evaluation of statistically significant data a *p*-value of *p* < 0.05 was chosen. Values are marked as following: *p* < 0.05 (^∗^) and *p* < 0.001 (^∗∗^). The differences between the three groups were accepted as significant if the area between the respective groups were at least two times increased or decreased, all other lipids are not further investigated. All data is presented as base 2 logarithm of the respective ratio. The respective standard error of the mean (SEM) was calculated for each group for all determinants.

In contrast to this, alkyl-/acylphosphoethanolamines (aPE) are mainly downregulated (six out of eight) in YM and OMU as compared to the OMI (see Figure 4). We detected seventeen aPEs in total, however, nine of them did not show any significant level change between groups. None of the aPEs showed statistically significant changes for the ratio of YM and OMU. Consequently, these lipids are supposed to be mainly related with impaired memory. In more detail, aPE 40:5 is the most downregulated lipid in YM and OMU in comparison to OMI. The lipids aPE 36:3, 38:3, and 40:8 were downregulated in YM only in comparison to OMI, whereas aPE 40:4 and 42:3 are upregulated in YM compared to OMI.

Furthermore, the levels of six out of seventeen detected phosphoethanolamines (PE) were found to be significantly regulated between groups (Table 1). In YM all six PEs were significantly upregulated in YM vs. OMI, whereas between YM and OMU no differences could be detected. PE 36:1 and 38:3 were found to be upregulated in OMU as compared to OMI.

#### Glycerophosphserines

The levels of glycerophosphoserines (PS), another main class of glycerophospholipids, were found to be regulated in association with aging and impaired memory (Table 1). In total, five PS (PS 36:1, 36:2, 38:4, 40:4, and 40:6) were identified, all being significantly increased in YM compared to OMI and with the exception of PS 36:1, also in comparison to OMU. The comparison between the aged groups revealed an increase of PS 36:1, 40:4, and 40:6 in the OMU over the OMI.

#### Triacylgycerols

Triacylglycerols (TG), a major compound class of glycolipids, seem to be only a minor building block of the hypothalamus as only two species could be detected: TG 52:3 and 52:4. Both of them showed a significantly increased level in YM compared to OMI. TG 52:3 is suggested to be linked to aging, as it is also upregulated in YM compared to OMU, whereas no significant differences between the aged groups could be detected. In contrast, TG 52:4 seems to be associated with impaired memory as it is significantly upregulated in OMU compared to OMI but similar between YM and OMU.

#### Ceramides

**FIGURE 5:**
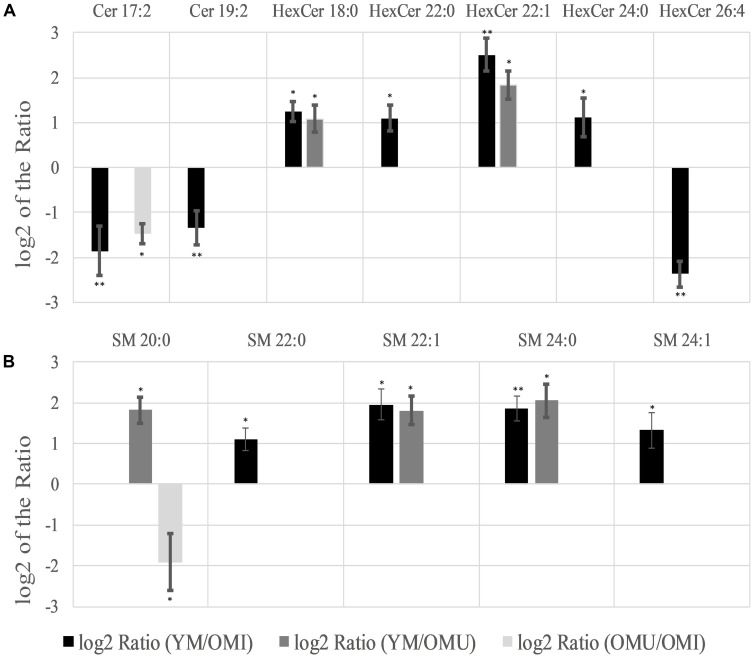
Significant level changes in sphingolipids [**(A)** Cer and HexCer; **(B)** SM] in the hypothalamus with either up- or downregulated levels concerning aging and impaired memory. Abbreviations: Cer, ceramides; HexCer, hexosyceramides; SM, sphingomyelins; YM, young male Sprague-Dawley rat; OMI, old male Sprague-Dawley rat impaired; OMU, old male Sprague-Dawley rat unimpaired. aPC, alkyl-/acylglycerophosphocholines; LPC, lysoglycerophosphocholines; PC, diacylglycerophosphocholines; YM, young male Sprague-Dawley rat; OMI, old male Sprague-Dawley rat impaired; OMU, old male Sprague-Dawley rat unimpaired. Statistics: Differential lipidomic analysis of YM, OMU and OMI rats were performed by SIEVE^TM^ software (version 2.1, Thermo Scientific) for peak detection and alignment into features followed by manual feature identification. For evaluation of statistically significant data a *p*-value of *p* < 0.05 was chosen. Values are marked as following: *p* < 0.05 (^∗^) and *p* < 0.001 (^∗∗^). The differences between the three groups were accepted as significant if the area between the respective groups were at least two times increased or decreased, all other lipids are not further investigated. All data is presented as base 2 logarithm of the respective ratio. The respective standard error of the mean (SEM) was calculated for each group for all determinants.

Significant regulations in ceramides (Cer) could also be found (Table 1 and Figure 5A). Two out of seven detected ceramides (Cer), namely Cer 17:2 and Cer 19:2, showed a statistically significant downregulation in YM compared to OMI. Cer 17:2 is suggested to be associated with impaired memory as it was also decreased in the group of OMU compared to OMI. Five out of thirteen detected hexosyceramides (HexCer), derivatives of Cer, showed significantly changed levels. Four HexCer were upregulated in YM compared to OMI, all containing short and mostly saturated chains (HexCer 18:0, 22:0, 22:1, and 24:0). HexCer 18:0 and 22:1 are also upregulated in YM compared to OMU and are therefore suggested to be mainly linked to aging. HexCer 26:4 possessing a longer polyunsaturated chain, however, was found to be increased in OMI compared to YM rats.

#### Sphingomyelins

Five out of eleven sphingomyelins (SM) showed level changes in at least one of the three groups (Figure 5B). Their acyl side chains possess between 20 and 24 carbon atoms and are either saturated or mono-unsaturated. SM 22:1 and SM 24:0 are proposed to be linked to aging as they are upregulated in YM as compared to OMI and OMU. However, no significant differences in the group of OMU/OMI could be detected. SM 22:0 and SM 24:1 were only upregulated in YM compared to OMI. SM 20:0 is the only lipid species which was found to be group dependently up (in YM compared to OMU)- or downregulated (in OMU as compared to OMI).

## Discussion

We found differences in the hypothalamic lipid contents especially when YM and OMI male rats were compared, whereas the number of lipids that differed between YM and OMU were clearly lower. The difference between OMI and OMU in the cognitive status suggests a contribution of hypothalamic lipid composition to spatial cognitive processes. Effects of age-related obesity and resulting dysregulation of cerebral blood flow (Tucsek et al., 2014) seem to be unlikely since we did not find a statistically significant difference in body weights between OMI and OMU. Also effects of consumed lipids (especially through high fat diets) on cerebrovascular functions (De Wilde et al., 2003) is improbable, since all groups received the same, energy reduced diet.

Studies regarding the effects of glycerophosphocholines upon cognitive processes are limited. Granger et al. (2019) found reduced levels of lyso-phosphatidylcholines (LPC (18:0/0:0; 16:0/0:0; 24:6/0:0; 25:6/0/0) as a result of reduced activity of cytosolic phospholipase 2 (A_2_) in all cortical regions, but not the hippocampus in water maze memory impaired mouse strains of Alzheimer’s disease (AD). A_2_ triggers the hydrolysis of phosphatidylcholines (LPC) as well as phosphatidylethanolamines (LPE) which removes one fatty acid group. This may be a cognition-lipid related mechanism also in the present study. On the one hand, we found a reduction of some glycerophosphocholines in OMI as compared to OMU, namely aPC 36:2, PC 34:0 and PC 36:2, while others are increased in the first group. L-alpha-glycerylphosphorylcholin (α GPC), a metabolite of phosphatidylcholin and a precursor of the neurotransmitter acetylcholine, is used as a cognitive enhancer in the therapy of AD and dementia (Lopez et al., 1991; Drago et al., 1992; Lee et al., 2017). In addition, a series of LPE species are reduced in OMI compared to OMU. This lipid group is best and consistently discriminated between OMI and OMU. LPE like other lipids play a role in cell signaling and activation of other enzymes (Piomelli et al., 2007). Inhibition of β secretase 1, an enzyme involved in the synthesis of β amyloid during AD disease, mainly restores the fatty acid composition of LPE and induce cognitive recovery in a mouse model of AD (Villamil-Ortiz et al., 2016).

Triacylglycerols do not substantially contribute to discriminate between OMI and OMU. Interestingly, Yetimler et al. (2012) also found no differences in hypothalamic levels of fatty acids between young, cognitively impaired and unimpaired aged mice tested in a water maze task.

Ceramides generally are related to impaired learning and memory. Decreased memory function in Parkinson patients was correlated with increases of plasma levels of Cer 22:0, Cer 20:0 and Cer 18:0 (Xing et al., 2016). Verbal memory performance was predicted by increased levels of Cer 22:0, Cer 24:0, and Cer 18:0. In the present study regarding the hypothalamus we found differences in only one ceramide (Cer 17:2) and the level was reduced in OMI as compared to OMU. Thus, levels of ceramides and sphingomyelins are less affected and cannot serve as biomarkers of cognitive decline in the hypothalamus in contrast to the hippocampus, where both lipid species predicted spatial cognition and cognitive flexibility (Huston et al., 2016; Maliković et al., 2019b).

Because the hippocampus is a central brain region involved in spatial learning and memory an influence of hypothalamic activity and signaling upon this structure is likely involved in spatial cognitive impairment in the present study.

In fact, Chamorro-López et al. (2015) found an improvement in memory of a spatial water maze task in rats with posttraining self-stimulation of the lateral hypothalamus accompanied with an increase of the structural plasticity of hippocampal cornus ammonis 1 (CA1) neurons as compared to sham operated rats without stimulation. Whether this is based on a direct effect upon hippocampal neurons, possibly *via* the septum/diagonal band (Albert-Gascó et al., 2017) or a more indirect mechanism *via* activation of the hypothalamic – pituitary – adrenal axis (HPA), which triggers the release of corticosterone in the adrenals remains to be investigated. Corticosterone easily passes the blood brain barrier and can be found in brain regions shortly after release. Corticosterone *via* the activation of corticosterone binding receptors, mainly glucocorticoid (GR) and mineralocorticoid (MR) receptors strongly regulate hippocampal neuronal and memory processes by fast non-genomic and slower genomic processes (McGaugh et al., 1975; Bohus and de Kloet, 1981; Joëls and de Kloet, 1991; Joëls, 2018). Fast processes are mediated by cell membrane bound and slower by intracellular receptors that translocate into the nucleus after activation, serving as transcription factors. Lee et al. found the HPA responses to stress is reduced in water maze memory impaired as compared to cognitively unimpaired aged rats. This was paralleled by a decrease of nuclear glucocorticoid receptors (GR) in the hippocampus (Lee et al., 2012). Nuclear receptors are considered to signal long-term genomic processes (Nishi, 2010) involved in long-term memory formation (Schwabe et al., 2010).

Recently, a regulatory interaction of thyroid hormones and corticosterone binding receptors in gene expression of mouse hippocampal cells probably related to learning and memory processes has been found (Bagamasbad et al., 2019). Spatial learning tests often induces task dependently milder or more severe stress responses. We found a certain increase in corticosterone (Uzakov et al., 2005; Schulz and Korz, 2010) as well as learning related regulation of corticosterone binding receptors in the hippocampus of male rats (Sase et al., 2014) in response to the here used hole-board paradigm.

Interestingly, in a recent prior study we found differences in steroid hormones in young male rats, OMU and OMI (Maliković et al., 2019a). Thyroid hormones, besides testosterone, dihydrotestosterone and androstanediol-3α17β, were specifically differently regulated between these groups. The TSH was the only one that discriminates between OMU and OMI. The hypothalamus, by release of the thyrotropin releasing hormone (TRH), is a pivotal activator of the release of TSH in the pituitary gland, which in turn improves cognition in elderly subjects widely unrelated to the subsequent release of triiodotyronin (T3) and thyroxin (T4) in the thyroid gland (Stwertka et al., 1992; Chachamovitz et al., 2016; Szlejf et al., 2018). TSH could be associated with better performance in a variety of cognitive tests in aged humans (Beydoun et al., 2012). Given the fact that lipids are strongly involved in cellular signaling related to learning and memory (Saleem et al., 2013; Huston et al., 2016; Xing et al., 2016) it may be feasible that the different hypothalamic lipid compositions and metabolism may be involved in the regulation of the release of TRH. Further neuroprotective effects of TRH itself has been reported in the hippocampus (Pizzi et al., 1999; Jaworska-Feil et al., 2001; Veronesi et al., 2007), the main brain structure involved in spatial learning and memory.

In summary, we found differences in a series of lipids in the hypothalamus of aged cognitively impaired and unimpaired male rats. Mainly and consistently, LPE were suitable to discriminate between these groups. Given the small body of literature regarding differences in hypothalamic lipid composition and age-related cognitive decline, this group of lipids should be specifically considered in further studies. Moreover, direct and indirect mechanisms of hypothalamic signaling by lipids in age related cognitive decline should be investigated.

## Data Availability Statement

All datasets generated for this study are included in the article/supplementary material.

## Ethics Statement

The animal study was reviewed and approved by Federal Ministry of Education, Science and Culture, Austria.

## Author Contributions

JW and VK have analyzed the data and written the manuscript. HK has performed the LC-MS analysis, performed database matching and calculated *p*-values. HH, AH, and DF have performed animal experiments. JL, GL, EU, and TL have contributed to data analysis, manuscript writing and conceptualized and supervised the experiments. All authors contributed to the article and approved the submitted version.

## Conflict of Interest

The authors declare that the research was conducted in the absence of any commercial or financial relationships that could be construed as a potential conflict of interest.
